# A Stronger Post-Publication Culture Is Needed for Better Science

**DOI:** 10.1371/journal.pmed.1001772

**Published:** 2014-12-30

**Authors:** Hilda Bastian

**Affiliations:** Scientist and Editor, National Center for Biotechnology Information, National Library of Medicine, National Institutes of Health, Bethesda, Maryland, United States of America

## Abstract

Hilda Bastian considers post-publication commenting and the cultural changes that are needed to better capture this intellectual effort.

*Please see later in the article for the Editors' Summary*

A research report or idea needs to clamber over more than the hurdle of publication to move science, practice, or policy forward. It's not only a matter of authors waiting for kudos and citations to roll in. If their work is not to sink into oblivion, or be acted on when it shouldn't be, publication is just the beginning. Both improving research quality [1],[2] and reducing waste in science [3] require a stronger post-publication culture.

Early Enlightenment science was rooted in ongoing discussion among scientists. Scientific discourse in a small, widely scattered community was in person and via books and the “erudite letters” that were the precursor of journal articles [4]. The journal system, capturing fragments of research, enabled massive expansion and acceleration of scientific activity [4].

These days the system does not keep up well with the speed of activity and the volume of research from a vast community. Articles are, by and large, too uncorrectable and unconnected [5], and much significant intellectual effort is not captured at all. Substantive discussions in journal clubs, in email lists, in social media, and at conferences are not distilled into a concise, permanent, accessible record. Most of the unaddressed content of pre-publication peer review is also lost.

Post-publication evaluation is highly fragmented. It often appears within future articles, either embedded in the introduction and discussion sections, or in formal research syntheses. Dedicated review journals (and journal sections) select, summarize, and critique publications, usually in an “expert picks” way. There are also rigorously structured systems of post-publication evaluation inside and outside journals [6],[7].

There are more immediate channels to respond to published research, such as letters and comments to the editor, commentaries, and editorials in journals, and discussion in blogs. Dedicated websites have been developed for discussing and sharing research among authors [8], and PubMed Commons (for which I am editor) enables post-publication commenting and linkages by the PubMed authorship community and journal clubs [9].

Somewhere within this activity is the amorphous phenomenon that people call post-publication peer review. For some, post-publication peer review is simply shifting pre-publication peer review to after an article's release [10]. For others, it's any evaluation of an article that is similar to pre-publication peer review. Post-publication peer review overlaps with post-publication commenting, but does not encompass all of that activity.

## Post-Publication Commenting

Many associate post-publication commentary with only the negative “yin” of criticism, correction, retraction, and failed replication. It is essential to prevent research-led error, harm, and futile studies. But there is a vital positive “yang” aspect, too, incorporating research aftercare [11]. Answers to questions may be critical for other studies, for adequate research assessment and synthesis, and for considering practice and policy implications [12]. Discussion can build, apply, connect, and update ideas and ongoing work.

For some, though, the success of post-publication commentary is concerned only with the “yin” of correction and retraction. For others, post-publication evaluation only “works” if it occurs for all articles, making pre-publication peer review redundant. From these perspectives, post-publication evaluation would always be shortchanged, and be seen to fall short. However, success includes rescuing important work from obscurity, and building work and capacity, not just tearing it down. Updating is at least as critical as correction to improving published research.

Furthermore, the scientific evidence base for effects of routine pre-publication peer review on article quality remains weak [13]. Pre-publication peer review can also worsen the quality of research, as when peer reviewers demand unplanned analyses of clinical trials [14]. With an oversaturation of publication in many areas, assessing it all only exacerbates the waste. Post-publication review faces the same problems.

## Cultural Challenges to Post-Publication Activity

**Figure 1:**
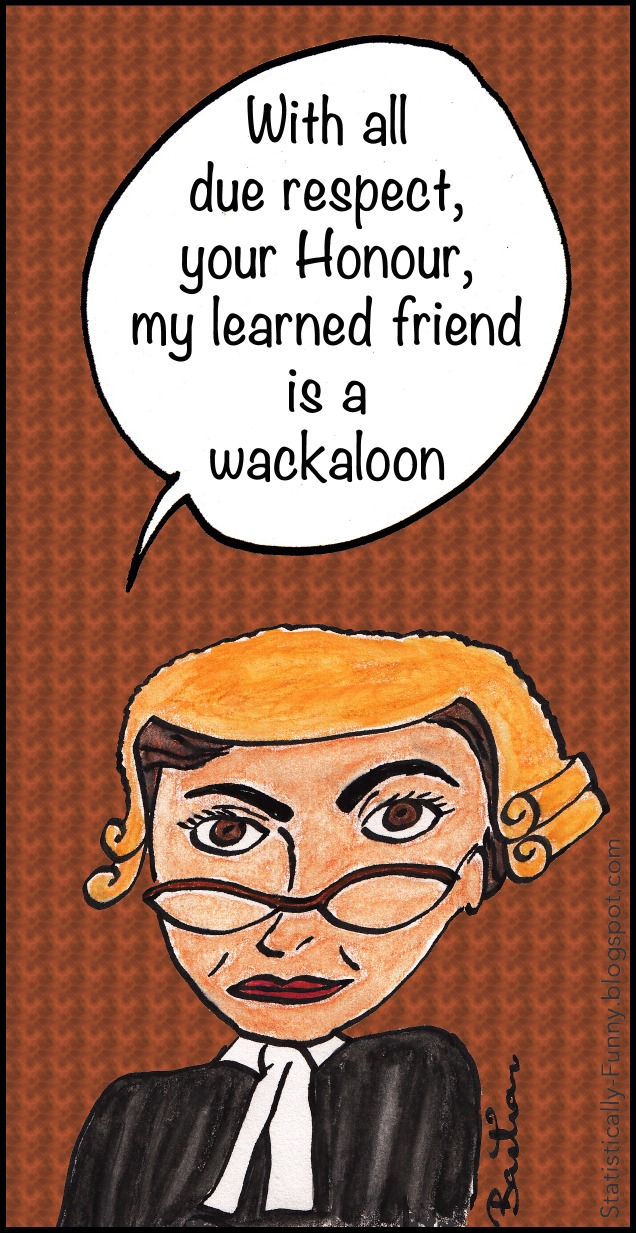
The melding of Internet culture and traditional communication. (Wackaloon: Internet slang for a kook; believed to derive from wacky and loon [33].)

Many are wary or worse about post-publication culture. For some, any un-peer-reviewed response to peer-reviewed work is impertinent, and the Internet's removal of constraints to adding both substantive and trivial post-publication commentary to the public space is hard to accept. The Internet has also increased the quantity of incivility out in public view (Figure 1).

Disputes between scientists have always been common, and it has always been the case that “in the bitter social conflict that ensues, the standards governing behavior deteriorate” [15]. According to sociologist Robert Merton, the example of Edmond Halley calling another astronomer a “lazy and malicious thief” in the 17th century was, and remains, more commonplace than aberrant [15]. He saw these conflicts as arising from the same “deep devotion to the advancement of knowledge” that fuels the passion for engaging in intellectual labor. We need to study and improve the way we communicate and cope with our errors and criticisms of our work.

The fear of repercussions for junior scientists in particular is high. This fear lies at the heart of the contentious issue of anonymous post-publication commenting. Some argue, though, that the risks for young scientists of openly commenting on others' work do not necessarily outweigh the advantages of visibility and recognition [16].

Even if the cost is reticence about participating, I believe the balance tips towards the requirement for transparency. Readers need to be able to judge whether writers are commenting outside their areas of expertise. Concerned readers need to have a chance of recognizing writers who have conflicts of interest, or be able to investigate whether or not potential conflicts exist.

However, addressing the obstacle to scientific progress posed by social dominance and aggression is a critical cultural issue, and not only—or even necessarily predominantly—for young researchers. Stereotype threat (anticipating discrimination) and other social issues may deter women scientists and other groups from commenting, too. Social influences can make women less talkative and less assertive than men in mixed gender groups [17], especially where “participants' concerns for self-presentation are heightened” [18].

Women scientists seem to be underrepresented in science activities that make their reflections public. In some fields and countries at least, women may still publish less [19]–[21], present less at conferences [22],[23], and blog less [24],[25]. A small body of research since the 1990s has identified some disturbingly low rates of participation by women as peer reviewers [26]–[28], though double-blind peer review might increase women's participation [29].

During the first year of PubMed Commons, less than 20% of those commenting were women. Research on gender bias in research and editorial peer review has been somewhat reassuring [26],[27],[30]. But the subject of this research has been the effect on publication fairness. The effect of under-participation on the development of confidence with the core science career skill of formulating valuable and effective critique was not considered.

I don't think that anonymity is a good solution. We need to consider skill development in critiquing research [13],[31]. That may also be valuable for those who are not scientific peers, but have contributions to make [32]. Developing a much more encouraging communication climate about errors and weaknesses of scientific communication is critical. This situation reminds me of the imperative identified decades ago to create a safety and quality culture in hospitals. A mature culture of responsiveness to complaints and problem identification is as much a prerequisite for research quality improvement as it was in health care.

Rewards for substantive intellectual effort post-publication and for the aftercare of research publications and sharing of data would help. Formal recognition is also necessary to undo the perverse incentive for authors to keep important insights and additional data until a subsequent publication. Such delays can last for months, if not years.

Passive consumption of scientific papers, and the withholding of adequate information by authors, cannot advance science. Thinking and talking about our responses to research reports is still science's vibrant and compelling intellectual core. Capturing that post-publication intellectual effort more rigorously is essential for better science.
